# Molecular and genomic insights into viral resistance in *Capsicum* spp.: pathogenesis, defense mechanisms, and breeding innovations

**DOI:** 10.3389/fpls.2025.1716114

**Published:** 2025-12-01

**Authors:** Jayabalan Shilpha, Won-Hee Kang

**Affiliations:** Department of Horticulture, Division of Applied Life Science (BK21 Four Program), Institute of Agriculture and Life Science, Gyeongsang National University, Jinju, Republic of Korea

**Keywords:** antiviral defense strategies, *Capsicum annuum*, dominant resistance, plant viruses, recessive resistance

## Abstract

Plant viruses represent a major challenge to agricultural systems, threatening global food security amid a rising population. Specifically, pepper cultivation (*Capsicum annuum* L.) is often hindered by various viral diseases, with more than 60 viruses identified as affecting pepper plants. The most efficient strategy for controlling viral diseases is the development of resistant cultivars of peppers. A comprehensive understanding of complex interactions between plant defense mechanisms and the strategies employed by viruses to evade these defenses, coupled with host factors that facilitate viral replication and movement, is essential for developing resistant cultivars. Natural antiviral defense mechanisms in plants are well characterized and include resistance genes, RNA silencing, autophagy-mediated degradation, translational repression, and resistance to viral movement. Recent advances in next-generation sequencing (NGS), genome-wide association studies (GWAS), high-density genotyping platforms and gene-editing tools such as CRISPR/Cas have accelerated the identification of resistance loci and key host factors involved in viral pathogenesis. This review summarizes current molecular and genomic insights into virus–host interactions in *Capsicum* spp., highlighting their role in advancing marker-assisted selection (MAS) and genomic-assisted breeding. The integration of molecular markers and genome editing into breeding pipelines offers new opportunities for developing durable, broad-spectrum viral resistance in peppers, ultimately supporting sustainable crop production and agricultural resilience.

## 1 Introduction

Plant viruses pose a significant threat to global food security and ecosystem services, damaging crops and cropping systems and resulting in an estimated annual global yield loss of approximately $30 billion and contributing to nearly half of all emerging plant diseases (Jones, 2021). Over 2,100 plant virus species have been officially recognized by the International Committee on Taxonomy of Viruses (Walker et al., 2021). The top ten viruses recognized for their widespread prevalence and significant global economic impact include *Tobacco mosaic virus* (TMV), *Tomato* sp*otted wilt virus* (TSWV), *Tomato yellow leaf curl virus* (TYLCV), *Cucumber mosaic virus* (CMV), *Potato virus Y* (PVY), *Cauliflower mosaic virus* (CaMV), *African cassava mosaic virus* (ACMV), *Plum pox virus* (PPV), *Brome mosaic virus* (BMV), and *Potato virus* X (PVX) (Scholthof et al., 2011).

Pepper (*Capsicum annuum* L.) holds significant economic value worldwide as a vegetable crop, primarily consumed for its fruits either in fresh or dried form, or utilized in the production of spicy condiments (Hernández-Pérez et al., 2020). According to FAOSTAT (2024), global pepper production increased steadily from 1.8 million tons in 1994 to 4.9 million tons in 2022, in both fresh and dried forms, highlighting its commercial significance (Kim et al., 2025). However, the commercial cultivation of pepper is significantly impacted by various environmental stresses, encompassing both biotic and abiotic factors (Kang et al., 2020; Kwon et al., 2021). Pepper plants encounter numerous plant pathogens, such as viruses, fungi, bacteria, nematodes, oomycetes, and viroids (Kang et al., 2022). The pepper cultivation across numerous regions worldwide is persistently hampered by a wide range of viral diseases. Approximately 68 viruses have been reported to infect pepper plants (Pernezny et al., 2003). Among them, about 20 virus species including both DNA and RNA viruses are notorious for causing substantial damage to pepper crops (Moury and Verdin, 2012). RNA viruses known to infect pepper plants include those from the genera *Cucumovirus, Tobamovirus*, *Polerovirus, Potyvirus* and *Crinivirus* (Kenyon et al., 2014). Among the DNA viruses, members of the genus *Begomovirus*, in the family Geminiviridae, are most commonly reported in pepper.

Viruses are obligate intracellular parasites that depend entirely on the host cellular machinery for replication and spread. Successful viral infection requires the coordinated manipulation of several aspects of the host cell biology, such as those that allow the virus to replicate its genome, suppress or evade plant defense mechanisms, and accurately transport the virus within and between cells (Medina-Puche and Lozano-Durán, 2019). The most well studied natural mechanisms of plant antiviral defense is mediated by resistance (*R*) genes (innate immunity), RNA silencing, autophagy-mediated degradation, translational repression, and resistance to virus movement (Akhter et al., 2021). The integration of diverse resistance genes via breeding initiatives, coupled with the application of genetic engineering and genome editing techniques like CRISPR/Cas technologies, shows significant potential in the development of economically valuable pepper crops, with enhanced resistance to viruses. Moreover, the advent of Next Generation Sequencing (NGS) technologies has facilitated more effective molecular breeding of pepper to combat both abiotic and biotic stresses, including devastating viral infections (Lee and Yeom, 2023; Kim et al., 2024b; Shilpha et al., 2024; Kwon et al., 2024a, Kwon et al., 2024b). These genomic resources facilitate the deployment of marker-assisted selection (MAS) and genomic selection (GS) for accelerating resistance breeding in pepper. Furthermore, the establishment of pangenomes, together with the use of specialized genetic populations such as recombinant inbred lines (RILs), introgression lines (ILs), and multiparent advanced generation inter-cross (MAGIC) populations, provides valuable frameworks for dissecting complex resistance traits and stacking multiple resistance genes to achieve durable and broad-spectrum viral resistance in *Capsicum* spp (Zohoungbogbo et al., 2024). This review provides a comprehensive overview of viral infection process and antiviral defense mechanisms in peppers, taking into account of viruses that commonly infect pepper crops. It also highlights recent molecular and genomic advances that offer new strategies for enhancing viral resistance in pepper breeding programs.

## 2 Viral factors contributing to pathogenesis in pepper

**Figure 1:**
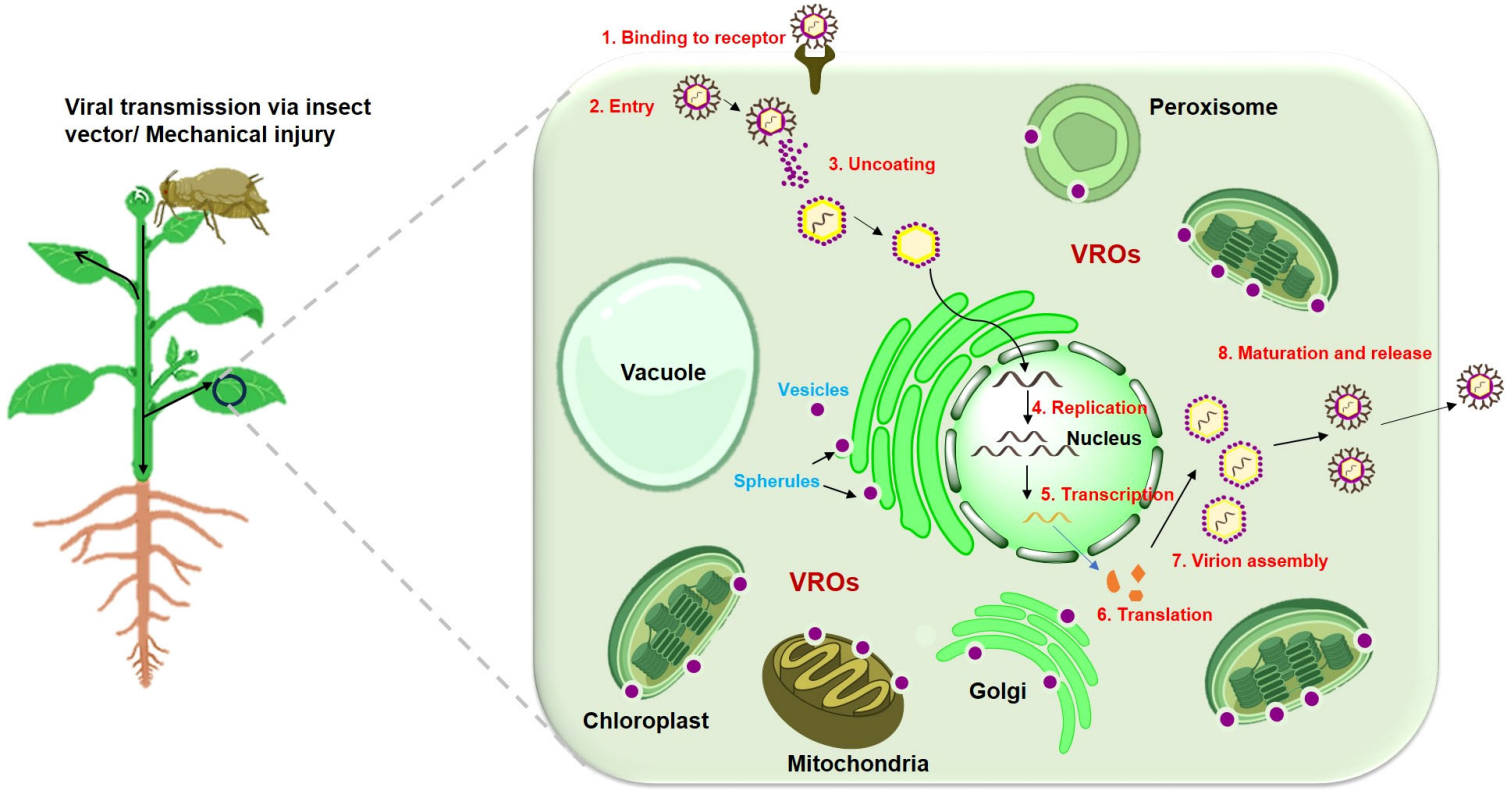
Schematic representation of viral transmission and replication within the plant cell. Virus enters the plant cell through an insect vector or mechanical injury. Once entered, the viral particle sheds its protective protein coat (capsid), releasing the viral genome (RNA or DNA) into the cytoplasm. Then, the viral genome is replicated using the host’s cellular machinery. For RNA viruses, replication occurs in the cytoplasm, whereas DNA viruses replicate using the plant’s nuclear machinery. Viral replication often involves the formation of specialized compartments or organelles to facilitate the replication process known as viral replication organelles (VROs). Specialized structures such as spherules and vesicles, often arise from the invagination of host cell membranes, such as the endoplasmic reticulum, chloroplast, mitochondria or peroxisomal membranes to facilitate viral replication and assembly. The viral genome then undergoes transcription and translation to produce viral proteins, including structural proteins, movement proteins, and proteins that suppress the plant's immune response. Once sufficient viral genomes and structural proteins are synthesized, they assemble into new viral particles (virions) in the cytoplasm. After infecting initial cells, the virus can spread systemically throughout the plant via the phloem, reaching distant tissues and organs.

Viruses, possessing a parasitic nature, have evolved adeptness in manipulating and hijacking cellular components, such as host proteins and organelles, to enhance their replication. Therefore, understanding how viruses manipulate cellular machinery to their infection cycles is crucial for developing plant varieties resistant to viral attacks. Upon entering the host cell, the viral genetic material – either DNA or RNA – serves as a template for transcription and translation processes, leading to the production of new viral genomes and proteins required for replication. While translation and replication selection occur in the cytoplasm, the actual replication process is confined to intracellular membranes, including chloroplast (Bwalya and Kim, 2023). Viral membrane proteins, direct the modification of host cellular membranes, leading to the formation of viral replication organelles/complexes (VROs/VRCs), the specialized membrane-bound viral replication compartments that facilitate the replication of the viral genome (Jovanović et al., 2023). These viral replication complexes are thought to restrict the viral replication process to a particular safe microenvironment that shields the replicating and progeny viruses from being attacked by host antiviral responses such as RNA silencing. To generate VROs, viruses typically target and remodel specific membrane-bound organelles such as the endoplasmic reticulum, chloroplasts, tonoplasts, peroxisomes, and mitochondria (**Figure 1**). Two categories of viral replication organelles or factories have been identified based on their morphology. These include spherules, which are invaginations of organelle boundary membranes, and tubular or vesicular organelles formed by host membrane protrusions (Nguyen-Dinh and Herker, 2021).

Nonsynonymous substitutions, which lead to amino acid changes in viral proteins, play a critical role in the evolution of viral pathogenicity. These mutations can profoundly affect the structure and function of viral proteins, thereby directly influencing their interactions with both viral and host factors. Based on the genetic variety brought about by amino acid alterations, plant viruses vary in their pathogenicity and degree of symptom severity on distinct host plants. For instance, the 13 Korean isolates of PepMoV were classified into two groups based on genetic variation, symptom severity, and pathogenicity on various host plants (Kim et al., 2008, Kim et al., 2009). By analyzing the ratio of synonymous (dS) to non-synonymous (dN) base substitutions in the P1 and 6K2 genes, as well as the amino acid (aa) variation that the 6K2 gene encodes, Kim et al. (2009) suggested that these genes may have a role in the pathogenicity and host specificity of PepMoV. Five resistance genes (*L^1^*, *L^1a^*, *L^2^*, *L^3^*, and *L^4^*) on chromosome 11 located at the *L* locus of the genus *Capsicum*, exhibits HR-mediated resistance against tobamoviruses such as PMMoV. Genda et al (2007) found that the *L^4^* gene-mediated resistance in *Capsicum chacoense* was overcome by two amino acid substitutions in the coat protein of PMMoV: glutamine (Gln) to arginine (Arg) at position 46 and glycine (Gly) to lysine (Lys) at position 85.

Specific amino acid substitutions are critical for systemic infection of CMV-Fny and CMV-P1 in peppers (Kang et al., 2012). The helicase domain of CMV RNA1 was identified as the key determinant of infection and virulence in peppers with *Cmr1* gene, which conferred resistance to CMV-Fny but not CMV-P1. Four residues (positions 865, 896, 957, and 980) in the CMV-P1 helicase domain were crucial for systemic infection, replication, and cell-to-cell movement. Subsequently, host genes such as formate dehydrogenase and calreticulin-3 precursor were identified to be interacting with the CMV-P1 helicase domain and crucial for CMV-P1 infection (Choi et al., 2016). Similarly, Han et al. (2020) reported four amino acid differences between PMMoV isolates ZJ1 and ZJ2 causing distinct chlorosis in pepper from Zhejiang, China. An Asn/Asp substitution in the coat protein determined chlorosis and localization: CP20Asp at the cell periphery, CP20Asn in chloroplasts. Fang et al. (2022) showed that specific residues in the HC-Pro and NIb-CP regions of PepMoV influence virus accumulation, movement, and symptoms. Tyrosine, glycine, and leucine at positions 360, 385, and 527 in HC-Pro enhanced accumulation/movement, while valine at position 2773 of NIb was crucial for symptom development. The CMV strain involved in mixed infection with tospoviruses is likely responsible for chlorosis in Indian hot pepper, as indicated by the amino acid substitution of Ser^129^ over conserved Pro^129^ in the coat protein (Vinodhini et al., 2021).

## 3 Anti-viral defense mechanisms in *Capsicum* species

In order to evade viral invasion, plants have evolved complex defense mechanisms that include RNA interference, RNA stability regulation, autophagy, protein breakdown via ubiquitination, hypersensitive response (HR), resistance responses mediated by *R* genes, and the induction of systemic acquired resistance (Lozano-Durán, 2024; Wei et al., 2024; Sharma et al., 2025). Plant resistance against viral disease is generally categorized into three types: non-host resistance, host resistance, and systemic acquired resistance (SAR) (Kang et al., 2005; Rai et al., 2022). Non-host resistance operates at the species level, where all members resist infection by a particular virus, often due to basal defenses, though the exact mechanisms remain unclear. Host resistance, also known as specific or cultivar resistance, involves specific genes in the plant that confer resistance to certain viruses, playing a crucial role in plant breeding. SAR occurs when plants activate defense mechanisms in response to pathogen exposure, leading to systemic protection and the activation of pathogenesis-related genes that produce antimicrobial compounds (Tian et al., 2024). This response is closely associated with the action of salicylic acid (SA) and can also be triggered by insect herbivory, thereby enhancing overall plant resistance.

### 3.1 Natural resistance mechanisms

Viral infection in plants involves several stages: entry into cells, uncoating of nucleic acid, translation of viral proteins, replication of nucleic acid, assembly of new virions, movement between cells, and spread to other plants (**Figure 1**). To prevent viral invasion, plants block viral movement at the cellular level, with some resistance mechanisms targeting viral replication or movement stages.

#### 3.1.1 Restriction of viral multiplication

Resistance at the single cell level, known as extreme resistance (ER) or cellular immunity, prevents virus replication within initially infected cells. The *C. annuum* cultivar 'Perennial' exhibited limited CMV multiplication in inoculated leaves, with a lower replication rate compared to the cultivars 'Vania' and 'Yolo Wonder' (Nono-Womdim et al., 1993; Lapidot et al., 1997). Additionally, 'Perennial' demonstrated resistance to other viruses, including various potyviruses (Caranta et al., 1996).

#### 3.1.2 Restriction of viral movement

When viruses infect plants, they spread from initially infected cells to neighboring ones and, for long-distance movement, through bundle sheath cells, phloem, and sieve elements. Plasmodesmata regulate both cell-to-cell and systemic movement. Plants counter by restricting viral spread, involving specific viral and host factors (Carrington et al., 1996). Resistance to CMV may involve limited entry, uncoating, restricted multiplication, or impaired long-distance movement, influenced by environment, CMV isolates, and host genetics (Chaim et al., 2001). In *C. annuum* varieties “Vania,” “Milord,” “L57,” and “L113,” resistance mainly limits long-distance CMV movement and is partial (Mihailova et al., 2013). Similarly, Indian chili “Perennial,” considered tolerant or partially resistant, restricts entry, reduces multiplication, and prevents systemic spread (Nono-Womdim et al., 1993; Caranta et al., 1997). In *C. annuum* cv. Avelar, PepMoV systemic movement is normally restricted but collapses under CMV co-infection, enabling PepMoV accumulation and systemic spread (Guerini and Murphy, 1999). In pepper, *pvr1* (*eIF4E*) was linked to PVY cell-to-cell movement (Ruffel et al., 2002). *C. frutescens* “BG2814-6” hinders viral replication and cell-to-cell spread (Grube et al., 2000), while *C. annuum* “Bukang” blocks CMV movement from epidermal to mesophyll cells (Kang et al., 2010).

### 3.2 Dominant resistance genes

**Figure 2:**
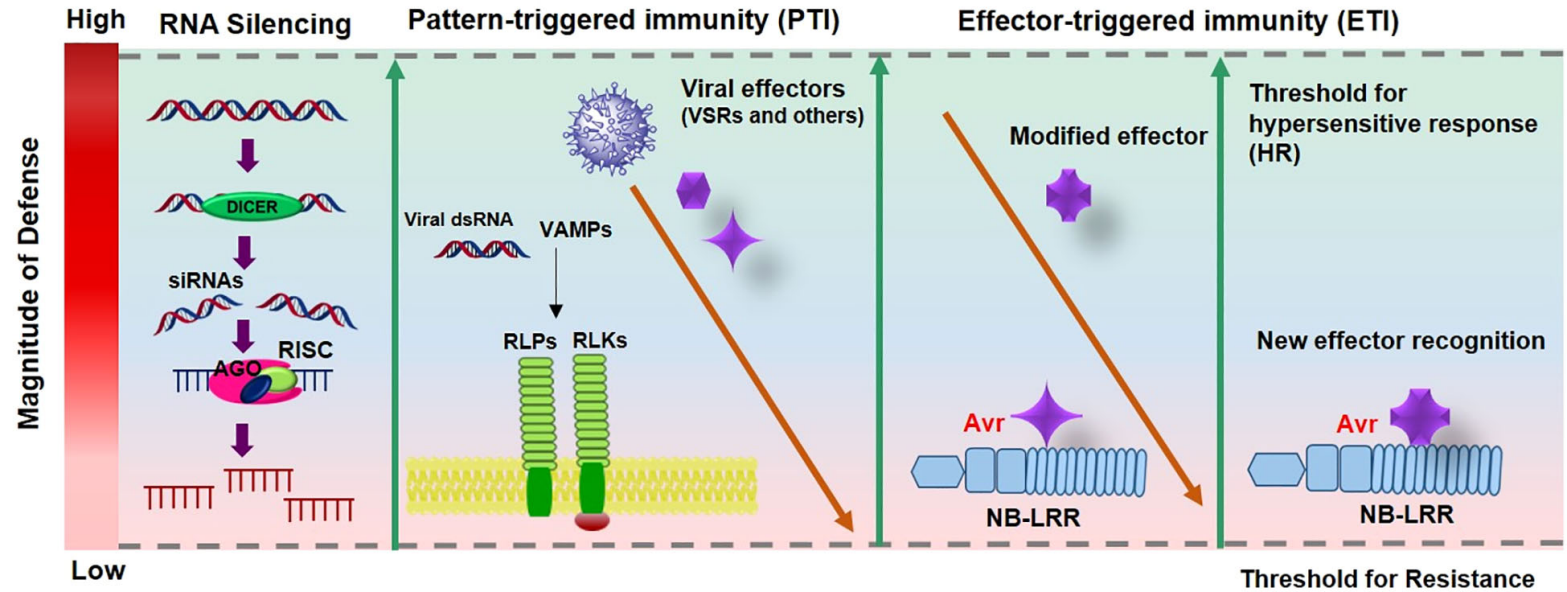
The zig-zag model [adopted from Jones and Dangl et al. (2006) and Piau and Schmitt-Keichinger, 2023)] illustrates the evolutionary combat between plants and viruses. In this model, silencing is often seen as a form of pattern-triggered immunity (PTI), where DICER like proteins detect Virus-associated molecular patterns, VAMPs, typically viral double-stranded RNAs. Additionally, PTI can play a role in antiviral defense through pattern recognition receptors (PRRs), functioning independently of RNA silencing. This initial defense is relatively weak and can be countered by viral effectors, including viral suppressors of RNA silencing (VSRs). A second, stronger layer of defense involves the specific recognition of viral effectors by NB-LRR proteins encoded by resistance (R) genes, leading to effector-triggered immunity (ETI). If strong and rapid enough, ETI can trigger hypersensitive cell death (HR). However, viruses may evolve modified effectors to evade recognition, prompting plants to develop new NB-LRRs capable of detecting the altered effectors and restoring ETI.

Dominant plant resistance (*R*) genes play a crucial role in antiviral defense, most of which encode nucleotide-binding site (NBS) and leucine-rich repeat (LRR) domains that enable recognition of pathogen-derived molecules and activation of defense responses (Collier and Moffett, 2009). In *Capsicum* and other plants, antiviral defense operates through two primary immune layers (**Figure 2**). The first layer, pattern-triggered immunity (PTI), is initiated by the perception of virus-associated molecular patterns (VAMPs) such as viral double-stranded RNAs by DICER-like proteins or membrane-bound pattern recognition receptors (PRRs) including receptor-like kinases (RLKs) and receptor-like proteins (RLPs). This basal immune response restricts viral replication and movement but is often suppressed by viral suppressors of RNA silencing (VSRs), which counteract RNA-based antiviral mechanisms and PTI responses.

The second, more specific layer, effector-triggered immunity (ETI), is activated when intracellular NB-LRR proteins recognize particular viral effectors (Avr factors). This recognition elicits a strong defense reaction, typically the hypersensitive response (HR), which confines viral spread by inducing localized cell death (Jones and Dangl, 2006). However, viruses continuously evolve modified effectors that escape recognition, leading to a dynamic co-evolutionary arms race where plants in turn develop new NB-LRR variants capable of detecting altered viral effectors and reinstating ETI (Piau and Schmitt-Keichinger, 2023).

In pepper, *Pvr4*, *Pvr7*, and *Pvr9* induce HR against potyviruses (Murphy et al., 1998; Janzac et al., 2009; Tran et al., 2015). The *Pvr7* from *C. chinense* PI159236 and *Pvr4* from *C. annuum* ‘CM334’ provide ER to PVY and PepMoV (Venkatesh et al., 2018) (**Table 1**). The *Pvr4* encodes a coiled-coil nucleotide-binding leucine-rich repeat (CNL) protein and confers resistance to PepMoV (Kim et al., 2017). The NIb protein of several potyviruses, including PepMoV, acts as an avirulence factor for *Pvr4*, inducing HR, whereas *Tobacco etch virus* (TEV) NIb does not due to lower sequence similarity (Kim et al., 2015). The *Pvr9*, similar to *Solanum bulbocastanum* Rpi-blb2, was identified via *Agrobacterium* transient expression on chromosome 6 and encodes a 1298-aa CNL protein. PepMoV slightly increases *Pvr9* expression in resistant ‘CM334’ but reduces it in susceptible ‘Floral Gem’. Both *Pvr4* and *Pvr9* mediate HR to PepMoV NIb (Tran et al., 2014, Tran et al, 2015). Resistance to PepMoV is linked to *Pvr4* and *Pvr7*; high-resolution mapping revealed *Pvr7* in *C. annuum* ‘9093’ is identical to *Pvr4* from ‘CM334’, representing the same locus (Venkatesh et al., 2018). *Tsw*, a dominant gene from *C. chinense*, confers HR to most TSWV isolates and is allelic with *Pvr4* at chromosome 10, sharing highly similar structures despite recognizing different viral effectors. The TSWV-NSs protein acts as a viral effector that triggers HR. The *Tsw* gene encodes CNL protein (Kim et al., 2017).

**Table 1:** Summary of identified virus resistance genes in *Capsicum annuum* L.

Resistance gene	Resistance type	Target virus	Virus family	Resistance mechanism /Description	References
*L^1^*–*L^4^*	Dominant	TMV, ToMV, PMMoV, TMGMV, BPeMV, ObPV, PaMMV, ToBRFV	Tobamovirus	NB-LRR proteins; stepwise race-specific resistance (*L^4^* most effective); partial resistance to ToBRFV	(Matsumoto et al., 2008; Tomita et al., 2011; Eldan et al., 2022; Poulicard et al., 2024; Ojinaga et al., 2024)
*pvr1*	Recessive	PVY, PepMoV, TEV	Potyvirus	Encodes *eIF4E*; blocks viral replication or movement; cell-autonomous resistance	(Murphy et al., 1998; Ruffel et al., 2002; Yoon et al., 2020)
*pvr2*	Recessive	PVY	Potyvirus	Mutated *eIF4E* allele; disrupts interaction with viral VPg protein	(Moury et al., 2004; Ruffel et al., 2006; Rubio et al., 2009)
*pvr2* alleles (*pvr2*^10^–*pvr2*^14^)	Recessive	PVY (e.g., PVY-F14K)	Potyvirus	New mutated *eIF4E* alleles with non-conservative amino acid changes; provides expanded resistance spectrum	Ibiza et al., 2010
*pvr3*	Recessive	PepMoV, partial to TEV/PVY	Potyvirus	Restricts systemic movement; accumulates in lower tissues but blocked above	(Murphy et al., 1998; Guerini and Murphy, 1999)
*Pvr4*	Dominant	PVY, PepMoV, PepSMV	Potyvirus	CNL protein; triggers HR via NIb protein (Avr); allelic to *Pvr7* and *Tsw*	Kim et al., 2015; Kim et al., 2017; Venkatesh et al., 2018
*pvr6*	Recessive	PVMV	Potyvirus	Encodes *eIF(iso)4E*; digenic recessive with *pvr2* for PVMV resistance	(Ruffel et al., 2006; Wang and Krishnaswamy, 2012)
*pvr6* alleles (*pvr6*²–*pvr6*^9^)	Recessive	PVMV, PVY	Potyvirus	Variants of *eIF(iso)4E* identified via EcoTILLING; provides potential extended resistance	Ibiza et al., 2010
*Pvr7*	Dominant	PepMoV, PVY	Potyvirus	Allelic to *Pvr4*; confers ER	Venkatesh et al., 2018
*Pvr9*	Dominant	PepMoV	Potyvirus	CNL gene (similar to Rpi-blb2); mediates HR in resistant lines	(Tran et al., 2014, Tran et al., 2015)
*Tsw*	Dominant	TSWV	Tospovirus	CNL protein; recognizes NSs protein as Avr; confers HR	(De Ronde et al., 2013; Kim et al., 2017)
*Cmr1*	Dominant	CMV (most strains)	Cucumovirus	It recognizes viral P1 helicase domain; provides strain-specific resistance	(Kang et al., 2010, Kang et al., 2012; Choi et al., 2016)
*cmr2*	Recessive	CMV (including CMV-P1)	Cucumovirus	Provides broad-spectrum resistance to CMV strains including CMV-Korean, CMV-Fny, and CMV-P1 (that had overcome *Cmr1*resistance).	(Choi et al., 2018)
*cvr4*	Recessive	ChiVMV	Potyvirus	Resistance linked to alternative splicing of DEM.v1.00021323; restricts viral spread	(Lee et al., 2025)
*bwvr*	Recessive	BBWV2	Fabavirus	The *bwvr* locus contained four candidate genes including DEM.v1.00035533 which encodes NPF1.2 nitrate transporter; may restrict cell-to-cell movement, or systemic movement.	(Kim et al., 2024a)

*Capsicum* spp. possess a series of *L* resistance genes that confer defense against Tobamovirus species and have been strategically incorporated into commercial cultivars to mitigate viral infections. These *L* genes encode NB-LRR immune receptors that mediate effector-triggered immunity (ETI) by recognizing specific amino acid motifs in viral coat proteins. This recognition induces conformational activation of the receptor, triggering downstream defense responses such as ion fluxes, reactive oxygen species (ROS) bursts, and transcriptional activation of defense genes, culminating in a localized hypersensitive response (HR) that restricts viral replication and movement (Poulicard et al., 2024). Four primary alleles: *L^1^*, *L^2^*, *L^3^*, and *L^4^* provide a stepwise, expanding spectrum of resistance (Tomita et al., 2011). The *L^1^* allele offers protection against P_0_ pathotypes such as TMV, *Tomato mosaic virus* (ToMV), TMGMV and *Bell pepper mottle virus* (BPeMV). The *L^2^* allele extends this resistance to include P_1_ pathotypes, including *Paprika mild mottle virus* (PaMMV), *Obuda pepper virus* (ObPV), TMV and TMGMV which can overcome *L^1^*-mediated resistance. *L^3^* confers resistance against P_0_, P_1_, and P_1,2_ pathotypes, such as those of PMMoV capable of overcoming *L^2^*. The *L^4^* allele further broadens this resistance to P_1,2,3_ pathotypes that can bypass *L^3^*-mediated resistance. Additionally, a temperature-sensitive allele, *L^1a^*, has been identified and may offer resistance under specific environmental conditions (Matsumoto et al., 2008)(**Table 1**). Despite these advances, the emergence of the P^1,2,3,4^ pathotype of PMMoV, capable of breaking *L^4^* resistance which underscores the urgent need for novel resistance genes and breeding strategies to counter this rapidly evolving pathogen (Ojinaga et al., 2024). A recent study revealed that pepper plants carrying *L* resistance alleles (*L^1^*, *L^3^*, *L^4^*) initiate a HR response to *Tomato brown rugose fruit virus* (ToBRFV) through recognition of its CP; however, this resistance was only partial, permitting transient systemic infection without the development of fruit symptoms (Eldan et al., 2022).

### 3.3 Recessive resistance genes

Recessive resistance genes arise from loss or alteration of critical host factors that viruses exploit for replication, translation, or movement. Such mutations prevent the virus from completing its infection cycle, offering a broader and more durable protection than dominant genes. They are particularly common against potyviruses, with many encoding eukaryotic translation initiation factors *eIF4E* or *eIF4G*, which are required for potyvirus RNA translation and replication complex formation. When these host factors are structurally altered or absent, the viral VPg protein fails to interact with them, thereby blocking viral RNA translation and replication. In *Capsicum*, *pvr1* and *pvr3* represent distinct loci conferring different resistance types to PepMoV (Murphy et al., 1998). For instance, *pvr1* present in *C. chinense* PI159236 and PI152225, encodes an eIF4E homolog whose amino acid substitutions disrupt VPg–eIF4E binding, providing broad resistance to PepMoV, TEV, and PVY (Yoon et al., 2020). In contrast, *pvr3* in *C. annuum* ‘Avelar’ restricts PepMoV differently; virus accumulates in inoculated leaves and vascular tissue but does not reach upper leaves. Co-infection with CMV disrupts this restriction (Guerini and Murphy, 1999).

Recessive genes *pvr2* and *pvr6*, on chromosomes 4 and 3, confer digenic resistance to *Pepper veinal mottle virus* (PVMV), though their effect on PepMoV is unknown (Ruffel et al., 2006; Rubio et al., 2009) (**Table 1**). The *pvr1* gene encodes an eIF4E homolog, and *pvr6* likely encodes eIF(iso)4E; mutations in these factors confer potyvirus resistance (Wang and Krishnaswamy, 2012). Ectopic *pvr1* expression in tomato imparts dominant resistance to PepMoV and TEV (Kang et al., 2007).

A novel recessive gene, *cvr4*, confers resistance to *Chilli veinal mottle virus* (ChiVMV), fine-mapped to a 2 Mb region on chromosome 11; functional validation identified DEM.v1.00021323 with resistance-associated alternative splicing (Lee et al., 2025). Similarly, *cmr2*, located on chromosome 8 in *C. annuum* ’Lam32’, confers broad resistance to CMV, including the virulent CMV-P1 strain, possibly by modulating host translation or replication pathways (Choi et al., 2018). Recently, *bwvr*, identified via bulked segregant RNA-seq, confers BBWV2 resistance and maps to a 116 kb region on chromosome 4 containing NPF1.2 (a nitrate transporter), which harbors a resistance-associated SNP that may alter virus–host transport dynamics (Kim et al., 2024a).

### 3.4 Avirulence genes

Viruses play a significant role in pathogenesis through both direct and indirect interactions. Plants possess *R* genes that provide defense against specific pathogens, leading to an incompatible interaction with viruses termed as avirulent pathogens. The pathogen molecule triggering this response is called the avirulence (Avr) determinant (**Figure 2**) (De Ronde et al., 2014). To identify these determinants, researchers often use reverse genetics, exchanging genome parts between viral DNA clones. Viral genes, including RNA polymerase subunits, movement proteins, and coat proteins, have been identified as avirulence factors. For example, the coat proteins of seven tobamoviruses infecting pepper such as TMV, TMGMV, PMMoV, ToMV BPeMV, ObPV, and PaMMV act as Avr factors, triggering resistance governed by their corresponding localization alleles (Tomita et al., 2011). The potyviral genome-linked protein (VPg) from potyviruses is a well-known avirulence factor associated with *pvr1*, *pvr2* and *pvr6* in *C. annuum* (Moury et al., 2004; Kang et al., 2005; Charron et al., 2008; Perez et al., 2012). VPg is a key protein in potyviruses that interacts with *eIF4E* and *eIF(iso)4E*. Mutations in these genes can disrupt this interaction, preventing viral replication (Ruffel et al., 2006; Liu et al., 2025).

Pepper harbors both dominant and recessive *R* genes against potyviruses. The dominant *Pvr4* gene (from *C. annuum* ‘CM334’) confers extreme resistance to multiple potyviruses including PepMoV, PVY and PepSMV. The Avr determinant for *Pvr4* is the viral RNA‐dependent RNA polymerase (NIb); single amino acid changes in NIb can abrogate *Pvr4* recognition (Moury et al., 2023). Kim et al. (2015) demonstrated that the RdRp NIbs proteins of PepMoV, PVY and *Pepper severe mosaic virus* (PepSMV), function as Avr factors, triggering *Pvr4*-mediated resistance in pepper plants.

Pepper resistance to *Cucumber mosaic virus* (CMV) is generally quantitative, but a major locus *Cmr1* (on chromosome 2) confers broad resistance to most CMV strains (Kang et al., 2010). The Avr factor for *Cmr1* has been mapped to the viral P1 helicase domain. The CMV‐P1 strain can overcome *Cmr1*, and Kang et al. (2012) showed that the RNA1‐encoded P1 helicase of CMV-P1 is necessary to infect *Cmr1* plants (Kang et al., 2012). In other hosts, CMV coat protein or 2b protein are sometimes recognized (e.g. tomato’s Ty genes), but in pepper P1 appears to be the key effector. As with other viruses, strain specificity is common: *Cmr1* confers resistance to some CMV isolates, while others (like CMV-P1) carry P1 variants that escape detection (Choi et al., 2016).

Pepper’s single dominant gene *Tsw* (from *C. chinense* PI159236) confers HR‐type resistance to TSWV (Kim et al., 2017). Recent work has identified the TSWV non‐structural RNA‐silencing suppressor protein NSs as the Avr elicitor for *Tsw* (De Ronde et al., 2013). Transient expression of NSs from a resistance‐inducing isolate triggers HR in *Tsw*‐carrying *Capsicum*, whereas NSs from a resistance‐breaking strain does not (De Ronde et al., 2013). This contrasts with tomato’s *Sw-5b* gene, which instead recognizes the TSWV movement protein NSm as its Avr effector (Huang et al., 2018). Thus, although pepper *Tsw* and tomato *Sw-5b* are orthologous NB‐LRRs conferring tospovirus resistance, they recognize different viral proteins, highlighting distinct resistance mechanisms and independent evolution.

### 3.5 Transcription factors

Transcription factors play a pivotal role in orchestrating antiviral defense in *Capsicum* spp., regulating hypersensitive response (HR) and defense gene expression through diverse mechanisms. While resistance (*R*) genes such as the *L* gene cluster directly recognize viral effectors to trigger HR, additional host factors, particularly transcription factors (TFs), play pivotal roles in amplifying and fine-tuning the immune response. These TFs mediate transcriptional reprogramming of defense-related genes, interact with signaling cascades such as mitogen-activated protein kinase (MAPK) pathways, and modulate hypersensitive and systemic acquired resistance (SAR) responses. Early studies identified the basic region-leucine zipper (bZIP) transcription factor PPI1 in *Capsicum chinense*, which is specifically induced by PMMoV infection and restricts viral replication and spread, underscoring the importance of pathogen-responsive bZIP TFs in defense signaling (Lee et al., 2002). Subsequently, members of the NAC family were shown to contribute to virus resistance. *CaNAC1*, a nuclear-localized NAC TF, is induced during incompatible interactions with PMMoV, promoting HR cell death and activating downstream defense genes (Oh et al., 2005). Similarly, *CaBtf3*, a NAC subunit, regulates transcription of defense-associated genes during HR and maintains proper protein folding under viral stress, further supporting antiviral responses (Huh et al., 2012a).

WRKY transcription factors have also emerged as key regulators of pepper antiviral immunity. *CaWRKYb* binds W-box elements in the *CaPR-10* promoter, positively regulating defense genes and enhancing resistance to TMV-P0; its knockdown reduces HR lesions and increases viral accumulation (Lim et al., 2011). *CaWRKYd*, a member of the WRKY IIa group, modulates TMV-mediated HR and PR gene expression, functioning as both a positive and negative regulator depending on context (Huh et al., 2012b). More recently, *CaWRKYa* was shown to be phosphorylated by TMV-responsive MAPKs (CaMK1 and CaMK2) and to enhance L-mediated resistance through transcriptional activation of *PR* genes, highlighting the integration of WRKY TFs with MAPK signaling pathways in virus defense (Huh et al., 2015).

## 4 Integrated genomic strategies for viral resistance in pepper

### 4.1 Genome based approach

In recent years, unprecedented advances in genomic resources and breeding technologies for pepper (*Capsicum* spp.) have accelerated the development of virus-resistant cultivars. Marker-assisted selection (MAS) has become a cornerstone of pepper breeding, particularly for traits governed by quantitative loci or recessive alleles and is complemented by advanced strategies such as marker-assisted backcrossing (MABC), recurrent selection (MARS), and pedigree selection (MAPS) to accelerate resistant cultivar development (Li et al., 2020a). Leveraging the extensive characterization of dominant and recessive resistance genes, along with their corresponding viral avirulence (*Avr*) genes, MAS has emerged as a highly effective tool for developing virus-resistant pepper cultivars. A wide array of molecular markers including SCAR, CAPS, KASP, SNP, and InDel markers has been developed for both dominant and recessive genes, such as the *L* gene cluster for PMMoV resistance, the *Tsw* gene for TSWV resistance, and the *Pvr* series for PepMoV resistance (Barka and Lee, 2020). Additional markers have been established for CMV (CaTm-int3HRM and InDel-2-134) (Kang et al., 2010; Guo et al., 2017) and *Pepper yellow leaf curl virus* (Chr-lcv-7 and Chr-lcv-12) (Siddique et al., 2022). These markers enable precise genotypic selection and integration of resistance loci into elite cultivars, supporting durable, broad-spectrum resistance.

Genome-wide association studies (GWAS) are a powerful tool for dissecting the genetic architecture of plant virus resistance. By scanning the genomes of diverse plant lines, GWAS identifies DNA markers, primarily SNPs, associated with susceptibility or resistance, pinpointing candidate genes and loci for breeding. Beyond detecting individual resistance genes or QTLs, GWAS can reveal interactions between loci, providing a comprehensive view of resistance mechanisms. For instance, Tamisier et al. (2020) examined over 260 pepper accessions for PVY response and identified seven SNPs on chromosomes 4, 6, 9, and 12, including two closely linked to the *pvr2* gene encoding eIF4E, while SNPs on chromosomes 6 and 12 overlapped with previously reported PVY resistance QTLs. Recently, Tamisier et al. (2022) conducted GWAS on 254 accessions for PVY response and identified a locus on chromosome 9 that controls systemic necrosis (tolerance) to PVY.

High‐quality reference genomes and pan‐genomes now capture the extensive structural variation in *Capsicum*; for instance, a graph‐based pan‐genome of 12 C*. annuum* accessions identified over 200,000 presence–absence variants (PAVs) and tens of thousands of copy‐number variants (Lee et al., 2022). Many of these PAVs co‐localize with loci associated with agronomic traits, including potyvirus resistance (Lee et al., 2022) offering a new reservoir of genetic diversity for breeding. The pepper pan‐genome has revealed many presence/absence variants in resistance gene analog clusters and other loci (Lee et al., 2022). For example, presence or absence of candidate NLR genes may underlie variations in potyvirus resistance among cultivars. By harnessing next-generation sequencing, Eun et al. (2016) applied genotyping-by-sequencing (GBS) to uncover two novel major QTLs conferring resistance to CMV isolate P1 (CMV-P1). Dense SNP maps and genotyping platforms (Fluidigm, GBS) are now used to screen diverse collections: recent surveys of thousands of accessions identified *C. chacoense* and *C. baccatum* germplasm carrying novel potyvirus and TSWV resistance alleles (Ro et al., 2024). Integrating these genomic tools with traditional QTL mapping and MAS enables breeders to pyramid multiple resistance genes and track desirable haplotypes efficiently.

Genomic selection (GS) further accelerates breeding by using genome-wide markers to predict plant breeding values for complex traits, including disease resistance. Although genomic selection has been successfully implemented in major crops such as wheat, soybean, and rice, its application in chili pepper remains limited, except for fruit-related traits in Korean accessions that exhibited high prediction accuracies (Hong et al., 2020). By integrating genotypic and phenotypic data, GS is expected to enable the efficient selection of superior progenies without extensive phenotypic evaluation, thereby facilitating the development of multi-virus-resistant pepper cultivars, particularly for quantitatively inherited resistances controlled by multiple minor QTLs.

Collectively, these genome-based approaches provide actionable targets for developing pepper cultivars that combine durable viral resistance with agronomically and commercially desirable traits, such as yield, fruit quality, and stress tolerance. By integrating MAS, GWAS, pan-genomics, and genomic selection, breeding programs can more effectively produce multi-virus-resistant *Capsicum* cultivars suited to diverse agro-climatic and market conditions, underscoring the transformative potential of genomics in modern pepper breeding.

### 4.2 Genome editing approach

**Figure 3:**
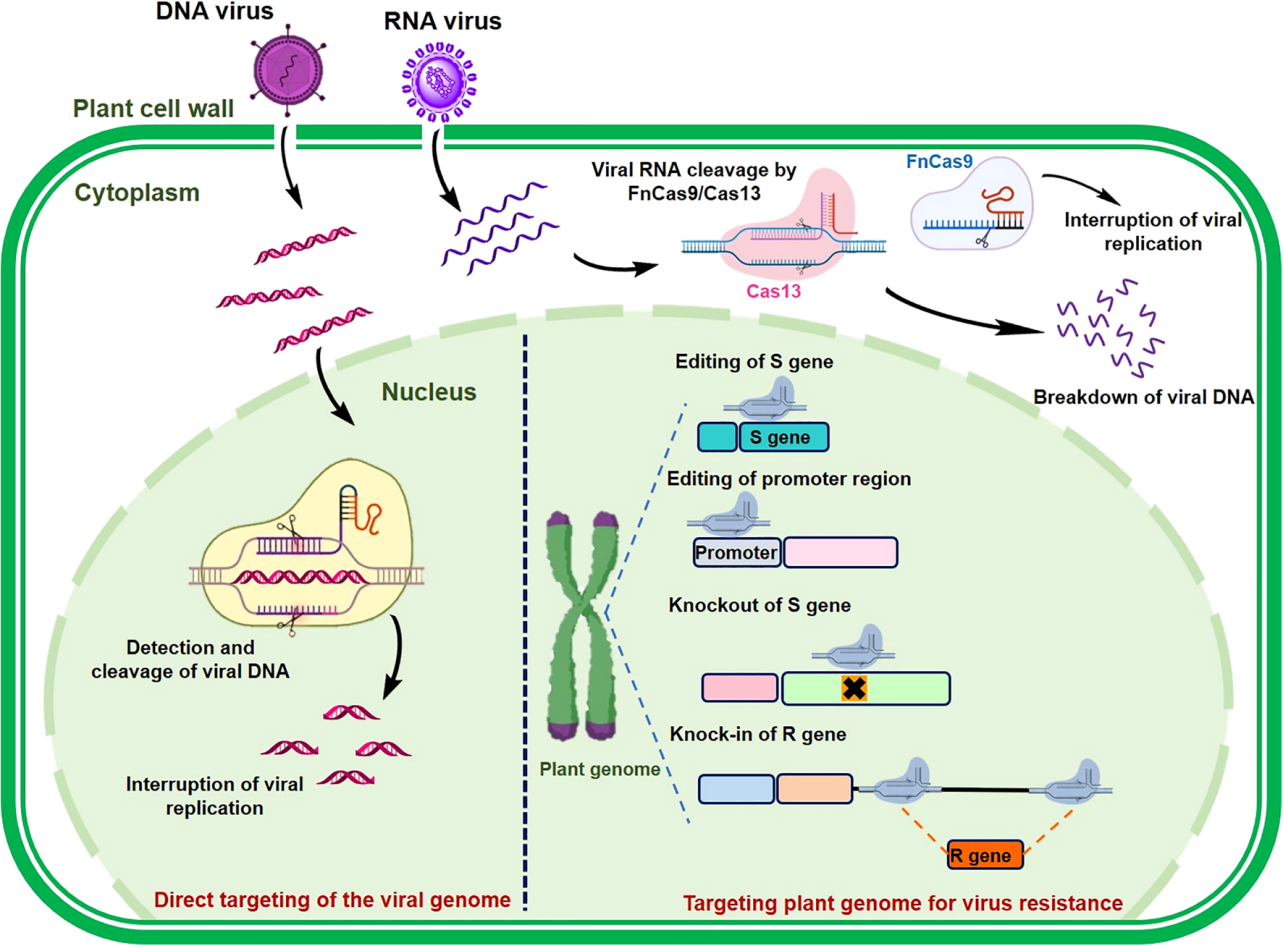
Class II CRISPR/Cas Systems for targeting viral and host genomes to engineer viral resistance in plants. When DNA viruses invade plant cells, the sgRNA-Cas9 complex recognizes and cleaves or alters the viral double-stranded DNA. For RNA viruses, both FnCas9 and Cas13a proteins, guided by their specific sgRNA or crRNA, effectively target and cut the viral genome. Additionally, CRISPR/Cas9 can modify host susceptibility factors, hindering viral infection by directly editing or knocking out plant susceptibility (S) genes, modifying promoter sequences to block pathogen-effector interactions, or facilitating the insertion of plant resistance (R) genes through homology-directed repair (HDR).

Recent breakthroughs in genome-editing technologies have provided powerful tools for precise modification of DNA sequences in plants. Among the genome-editing methods, CRISPR/Cas9 is distinguished by its remarkable efficiency, accuracy, and versatility, making it a preferred choice for plant genome editing and gene regulation (Jaganathan et al., 2018). CRISPR/Cas technology has been effectively employed to develop plant resistance to viral infections through various approaches, including plant-mediated resistance, alteration of host factors critical for viral entry, and direct manipulation of viral genomes (**Figure 3**). In plant-mediated resistance, the CRISPR/Cas system modifies host factors essential for viral infection, rather than directly targeting viral DNA or RNA. This is accomplished by disrupting host susceptibility genes (*S*-genes), which play a role in viral infection. In contrast, virus-mediated resistance focuses on editing the viral genome itself, with CRISPR/Cas systems precisely targeting and cleaving viral genetic material. CRISPR/Cas9-based gene editing can directly modify *S*-genes to develop virus-resistant crops. Although research on genome editing for virus resistance in peppers is currently lacking, this strategy has been successfully applied in other crop varieties, conferring resistance to a broad range of viruses, including those that infect peppers. As outlined in **Table 2**, genome-editing strategies targeting both viral and host factor genes in other crops provide valuable insights to guide future efforts in developing virus-resistant pepper cultivars.

**Table 2:** Application of genome editing techniques to confer resistance against pepper-infecting viruses in other crops.

Host plant	Virus	Editing system	Cas9 effector protein	Type of target	Target gene	Outcome	References
Tobacco/Arabidopsis	CMV, TMV	CRISPR/Cas9	FnCas9	Various regions of viral RNA	ORF1a, ORFCP and 3′-UTR	Reduced viral RNA accumulation and inheritable resistance to CMV and TMV in transgenic plants	Zhang et al., 2018
Potato	PVY	CRSIPR/Cas13	LshCas13a	Viral factors, replicase and capsid protein genes	P3, CI, NIb, CP	Transgenic potato lines expressing Cas13a/sgRNA constructs exhibited reduced PVY accumulation and disease symptoms.	Zhan et al., 2019
Tobacco	ChiLCV	CRISPR/Cas9	spCas9	Viral DNA replication and coat protein genes	C1/C4+V1/V2 C1/C4+IR C1/C4+V1/V2+IR	Multiplexed gRNA based CRISPR-Cas9 approach has efficiently reduced the viral titer and disease symptoms	Roy et al., 2019
Tomato	PepMoV	CRISPR/Cas9	spCas9	Host factor (translation initiation factor)	*eIF4E1*	Site-specific mutation of tomato *eIF4E1* conferred enhanced resistance to PepMoV	Yoon et al., 2020
Cherry tomato	PVMV	CRISPR/Cas9	SpCas9-NG	Host factor (translation initiation factor)	*eIF4E2*	The plants knocked out of *eIF4E2* exhibited complete resistance to PVMV-Ca31, partial resistance to PVMV-IC, and full susceptibility to the two PVY isolates tested, N605 and LYE84.	Kuroiwa et al., 2022
Potato	PVY	CRISPR/Cas9	spCas9	Host factor (translation initiation factor)	*eIF4E*	Mutated *eIF4E* lines showed enhanced resistance to PVY strain and were unable to interact with VPg	Noureen et al., 2022a
Potato	PVY	CRSIPR/Cas13	LshCas13a	Viral membrane protein, cytoplasmic laminate inclusion and viral genome linked protein genes	PI, HC-Pro, P3, Cl1, Cl2, and VPg	The transgenic potato plants expressing CRISPR/Cas13a with multiplex gRNA cassettes targeting conserved PVY genes exhibited broad-spectrum resistance against multiple PVY strains. Resistance efficiency was positively correlated with Cas13a/sgRNA expression.	Noureen et al., 2022b
Tobacco	PVY	CRISPR/Cas9	spCas9	Host factor (translation initiation factor)	*eIF4E1*-S, *eIF4E1*-T, *eIF4E2*-S and *eIF4E2*-T	Quadruple mutants harbouring loss-of-function mutations in *eIF4E1*-S, *eIF4E1*-T, *eIF4E2*-S and *eIF4E2*-T showed heritable high-level resistance to PVY in tobacco	Le et al., 2022
Tomato	PVY	CRISPR/Cas9	spCas9	Host factor (translation initiation factor)	*SleIF4E1*, *SleIF4E2*	Two amino acid substitutions, 119^H/Y^ and 123^S/N^, were identified in the viral-encoded VPg gene in SleiF4e1/e2 double mutant plants and exhibited an enhanced resistance to PVY	Kumar et al., 2022
Cherry tomato	Multiple potyvirus isolates	TALEN®, CRISPR/Cas9 and Cytosine Base editors	nCas9	Host factor (translation initiation factor)	*eIF4E1*	The plants carrying targeted mutations in *eIF4E1* showed resistance to multiple potyviruses when amino acid substitutions were introduced in both critical regions of the gene, whereas editing only one region failed to confer resistance.	Kuroiwa et al., 2023

Despite the availability of whole-genome sequences and genome-editing tools for peppers, the precision gene editing of peppers remains in its infancy, primarily due to the absence of a reliable transformation method. Although the limited morphogenic response of pepper explants is often seen as a key obstacle, other factors such as efficient DNA transfer and integration, along with the selection of recipient cells capable of regeneration, are also critical for successfully producing transgenic plants. Li et al. (2020b) were the first to pinpoint genome-wide CRISPR/Cas9 editing sites in pepper using the ‘Zunla-1’ reference genome. They further evaluated the specificity of these editing sites through a whole-genome alignment analyses. This study provided an essential groundwork for advancing CRISPR/Cas9-mediated gene editing in pepper. Concurrently, Kim et al. (2020) developed a stable DNA-free screening system for gene editing in hot pepper (‘CM334’) and sweet pepper (‘Dempsey’) by targeting the *CaMLO2* gene, linked to powdery mildew susceptibility, using CRISPR/Cas9 and Cpf1 (LbCpf1) systems. They demonstrated effective gene editing through PEG-mediated delivery of RNP complexes into protoplasts, establishing ‘Dempsey’ leaf protoplasts as a reliable platform for validating CRISPR tools and enhancing disease resistance in pepper plants. Subsequently, they developed a stable *Agrobacterium*-mediated gene editing method using the CRISPR/Cas9 system in the same pepper cultivars, using callus cultures to target the *CaMLO2* gene (Park et al., 2021). Recently, a biolistic method for delivering CRISPR/Cas9 reagents into peppers has been established, utilizing a construct containing two distinct guide RNAs that target the *phytoene desaturase* (*CaPDS*) gene (Bulle et al., 2024). Currently, virus-induced gene editing (VIGE) system leverages viral vectors to deliver gene-editing tools, such as CRISPR/Cas9, directly into plant cells (Daròs et al., 2023). This method utilizes the natural ability of viruses to infect plants, allowing for efficient and targeted editing of genes. More recently, two VIGE strategies have been developed using *Tobacco rattle virus* (TRV) and PVX vectors to target the *CaPDS* gene in Solanaceous crops, including tomato, potato, and eggplant (Lee et al., 2024), Liu et al (2023) established a transient CRISPR/Cas delivery system utilizing engineered TSWV vectors, which facilitates efficient somatic gene editing across various crop species, including pepper. Subsequently, they expanded their research to successfully generate transgene-free pepper plants, achieving high somatic editing frequencies of 57.65% and 75.73% at the *CaPDS-3* and *CaPDS-4* target sites, respectively, using TSWV vectors (Zhao et al., 2024). Thus, continued advancements in genome editing technologies, coupled with established transient and stable transformation methods will facilitate the successful development of virus-resistant pepper varieties in the future.

## 5 Discussion and future directions

Moving forward, enhancing viral resistance in *Capsicum* spp. will require the strategic integration of conventional breeding approaches with cutting-edge genomic and biotechnological innovations. Recent progress in next-generation sequencing (NGS), genome-wide association studies (GWAS), and high-throughput SNP genotyping have facilitated the identification and fine mapping of resistance genes or QTLs, enabling the development of reliable markers for marker-assisted selection (MAS) and genomic selection (GS). Tools such as *R* gene enrichment sequencing (RenSeq) have revolutionized high-throughput screening of germplasm and mutants, allowing for the rapid identification of candidate *R* genes recognizing key effectors (Jupe et al., 2014). To enhance the durability and broaden the resistance spectrum, strategies such as stacking multiple *R* genes are being explored. Furthermore, the development of RNA interference (RNAi)-based biopesticides is emerging as a targeted strategy to manage plant viruses, with the potential for effective application through suitable delivery mechanisms. The CRISPR-Cas system, a powerful genome-editing tool, presents additional advantages over RNAi approaches, particularly with the recent discovery of RNA-targeting CRISPR-associated proteins. However, the application of CRISPR-Cas system for engineering virus resistance in *Capsicum* is still in its early stages, constrained by transformation efficiency and the need for optimized delivery systems.

Interestingly, several resistance loci and resistance gene clusters identified in *Capsicum* are located near or within to QTL regions controlling fruit morphology traits such as fruit size and shape. For instance, the genomic region of the *Pvr4* gene on chromosome 10 is positioned adjacent to the fruit shape locus–associated QTL *fs10* (Borovsky et al., 2022) suggesting a potential co-evolution or genetic linkage between viral resistance and fruit development pathways. Moreover, transcription factors such as *CaWRKY*, which play roles in antiviral defense, have also been implicated in the regulation of fruit ripening and fruit maturation in pepper (Huh et al., 2012b; Cheng et al., 2016). These studies highlight the interconnected between defense and developmental networks in *Capsicum*. Integrating such loci into breeding pipelines through marker-assisted selection (MAS) and genomic selection (GS) will facilitate the development of high-yielding, virus-resistant cultivars with desirable fruit characteristics, thereby bridging molecular insights with practical advances in pepper improvement.

Future research should focus on validating the genomic regions and candidate genes identified through GWAS, pan-genome, and transcriptomic analyses in diverse genetic backgrounds. Functional validation using gene editing, mutant screening, and transcriptomic profiling under viral challenge will be essential to confirm their roles in conferring resistance. Additionally, the translation of these genomic discoveries into field-ready breeding programs through marker-assisted backcrossing, genomic selection, and genome-edited lines will bridge the gap between laboratory findings and practical cultivar development. Collaborative and multi-disciplinary breeding initiatives that integrate genomic insights, molecular validation, and market-oriented trait selection will not only enhance the durability of resistance but also accelerate the adoption of virus-resistant cultivars with improved consumer and commercial acceptance. Ultimately, the seamless integration of genomic innovation with traditional breeding will enable the development of resilient *Capsicum* varieties, fortifying global pepper production against future viral threats.
